# The effect of anaesthetist grade and frequency of insertion on epidural failure: a service evaluation in a United Kingdom teaching hospital

**DOI:** 10.1186/1471-2253-15-5

**Published:** 2015-01-21

**Authors:** Thomas P Heinink, Benjamin G Baker, Victoria F Yates, Dorothea C Addison, John P Williams

**Affiliations:** Department of Anaesthesia, Royal Derby Hospital, Uttoxeter Road, DE22 3NE Derby, UK; Division of Medical Sciences and Graduate Entry Medicine, School of Medicine, University of Nottingham, Royal Derby Hospital, DE22 3DT Derby, UK; MRC/Arthritis Research UK Centre for Musculoskeletal Ageing Research, University of Nottingham, NG7 2UH Nottingham, UK

**Keywords:** Analgesia, Epidural: standards, Statistics and numerical data, Utilization, Analgesia: surgery, Organization and administration

## Abstract

**Background:**

Despite being a commonly performed procedure, epidural catheter insertion has a significant failure rate. There is a lack of guidance as to how regularly the procedure should be performed in order to maintain competence. This study aimed to quantify whether increasing frequency of practice is associated with a reduction in failure rates.

**Methods:**

Data were collected prospectively on all patients undergoing intra-abdominal or thoraco-abdominal surgery who received epidural analgesia as part of their post-operative analgesic regimen over a 36 month period. Records were examined to identify the reason for epidural catheter removal, classified according to standardised definitions, the seniority of the inserting anaesthetist, and whether or not they were a permanent member of the anaesthetic department. Data were analysed using independent t tests, Mann–Whitney tests and Fisher’s test.

**Results:**

881 epidurals were inserted during the study period. 48 hour failure rate was 27.2%, whilst by 96 hours 33.9% of epidurals had failed. Increasing frequency of epidural insertion did not show a significant decrease in failure rate at either 48 (p = 0.36) or 96 hours (p = 0.28). However, long-term survival of epidurals at 96 hours was greater if inserted by permanent rather than temporary members of staff (non-permanent 60/141, 42.6% vs permanent 228/715, 31.9%, OR 1.58 (CI 1.09-2.29) p = 0.02).

**Conclusion:**

This study demonstrates that failure rates for postoperative epidural analgesia in major surgery are not dependent upon the frequency with which practitioners insert epidural catheters. However, failure rates are dependent on permanency of anaesthetic staff. These findings are significant when placed in the context of the General Medical Council’s requirements for clinicians to maintain competence in their clinical practice, suggesting that institutional factors may have greater bearing on epidural success or failure than frequency of task performance.

## Background

Each year approximately two hundred and thirty million major surgical procedures are undertaken worldwide [1]. Unfortunately pain is still common following these procedures with over three quarters of patients complaining of pain postoperatively and more than 10% complaining of severe pain in the immediate post-operative period [2]. Several meta-analyses and large randomized control trials indicate that epidural analgesia is associated with reductions in pain and morbidity in the post-operative period and, although the effect on mortality is less certain [3–6], consequently epidural analgesia is considered by many to be the gold-standard in providing analgesia following major surgery [7].

Despite being a commonly performed procedure, epidural failure remains significant, with published case series reporting failure rates, dependent on definition, of between 13-49% in the non-obstetric population [4, 8]. Previous studies have attempted to assess the reasons for this high failure rate, highlighting technical aspects of procedure performance such as insertion technique, method for securing the catheter and choice of drug administered [9, 10]. Researchers have also investigated the learning curve experienced by anaesthetic trainees for epidural insertion, with recently published studies reporting an increased failure rate for junior trainees compared to their more experienced peers in an obstetric setting [11]. Moreover studies have attempted to quantify the minimum number of procedures required for trainee competence, reporting successful catheter placement in 60% of cases after 25 insertions [12], and a 100% success rate after 80 attempts [13].

The General Medical Council (GMC) in the United Kingdom in their publication *Good Medical Practice*, state that doctors “must be competent in all aspects of their work” and “must regularly take part in activities that maintain and develop their competence” while they “must make clear the limits of their competence and knowledge” [14]. The American Medical Association, have published similar guidance, stating that doctors will “maintain technical proficiency in the clinical skills relevant to their practice” and “ensure that their scope of practice remains within their competence” [15]. Consequently in the absence of specific guidance from regulatory bodies concerning the number, regularity and quality of interventional procedures a clinician should perform, it remains unclear as to the number of epidural insertions required to achieve and maintain competence once trained.

Therefore in order to better understand the relationship between epidural performance and competence, we interrogated audit data collected over a three year period in our institution documenting the rate of epidural failure, as assessed by predefined criteria in patients who had undergone intra-abdominal surgery. This evaluation was performed to investigate whether failure rates were comparable to previously published series and to assess whether increasing operator seniority or frequency of insertion was associated with increasing success rates.

## Methods

This prospective evaluation of the epidural analgesia service in identifying causes of epidural failure was approved as a service evaluation by Mr Peter Korczak, chairman of the local ethics committee (National Research Ethics Service - Derby), who confirmed that neither ethical approval or informed consent from participants was required.

Patients receiving epidural analgesia as part of their post-operative analgesic regimen following intra-abdominal or thoraco-abdominal surgery were studied. Epidural catheters were inserted using a landmark technique (either loss-of-resistance to saline or `hanging-drop` technique). Whilst the initial loading dose of local anaesthetic +/− opioid adjuvant was administered at the discretion of the attending anaesthetist, a standard post-operative infusion, comprising 0.1% bupivacaine and 2 micrograms/ml fentanyl infused at a rate of between 0-15 ml/hr, was administered to all patients. Patients also received regular intravenous or oral paracetamol (4 g/day) and oral ibuprofen (1200 mg/day), if not contraindicated. All patients were nursed in a level 1 or 2 area, by nurses experienced in caring for patients with epidural catheters in-situ, and where vasopressor infusions could be used to treat hypotension.

Data were collected by specialist nurses from the acute pain service, on all patients receiving epidural analgesia over a 36 month period between February 2010 and February 2013. Patients were visited by a member of the acute pain service on a daily basis, from the first post-operative day and for the next 96 hours or until epidural catheter removal. These visits formed part of the usual post-operative care of patients ensuring adequate analgesia and epidural function, allowing for identification of epidural-related side-effects, and monitoring for rare complications such as hematoma or abscess formation. Data were not collected on patients who had epidural catheters removed before their return to the ward, and as such, no data were captured for patients who had immediate epidural failure, requiring the catheter to be removed in the recovery room.

Source data were collected from the anaesthetic chart, epidural care record and drug prescription card. The anaesthetic chart was examined to identify the vertebral level at which the catheter was sited and the grade of anaesthetist who inserted the epidural catheter, (classified as consultant; trainee; specialty doctor; or locum). Consultants and specialty doctors were considered to be permanent members of the anaesthetic department, whereas trainees and locums where considered to be temporary. Consultants were defined as doctors on the GMC’s specialist register and holding a substantive appointment at the trust. Locums were defined as doctors on the specialist register not holding a substantive post and employed by the Trust in a temporary capacity. Specialty doctors are practitioners with at least 4 years of postgraduate medical training, at least 2 of which are in anaesthesia [16]. Specialty registrars are doctors who have completed at least 2 years of training in anaesthesia and who hold a national training number.

Following wound inspection surgical incision was classified as above the umbilicus, below the umbilicus or both. The epidural care record was examined to identify the duration catheters remained in-situ post-operatively and reason for catheter removal.

Reason for epidural removal was classified as:

1. Removal as part of planned care (e.g. no longer required, removed at request of patient, surgeon or anaesthetist)

or

- Epidural failure, which was further classified as
- Technical failure (e.g. blocked catheter, inadvertent disconnection, leakage)
- Unacceptable side effects (e.g. hypotension, bradycardia, pruritus)
- Inadequate analgesia. Defined as; moderate to severe pain (5+ on an 11 point numeric rating scale) at rest persisting for two hours or more despite clinician intervention (e.g. bolus, catheter adjustment).

The amount of opioid analgesia used in the 24 hours following catheter removal was also recorded from the patient’s drug prescription card and converted to intravenous morphine equivalent using the British National Formulary standard conversion ratios [17].

All data were fully anonymised so that neither individual patients nor anaesthetists could be identified. Each anaesthetist was assigned a numeric code to allow individual practice to be monitored. Data were analysed using IBM SPSS Statistics software version 21 (Armonk, New York, USA). Distribution of data was tested using Kolmogorov-Smirnov test, with normal data expressed as mean (SD) and non-normal data as median ± interquartile range. Independent t tests were applied to plausibly normal data and Mann–Whitney tests to non-normal data. Categorical values were compared using Fisher’s test.

## Results

**Figure 1:**
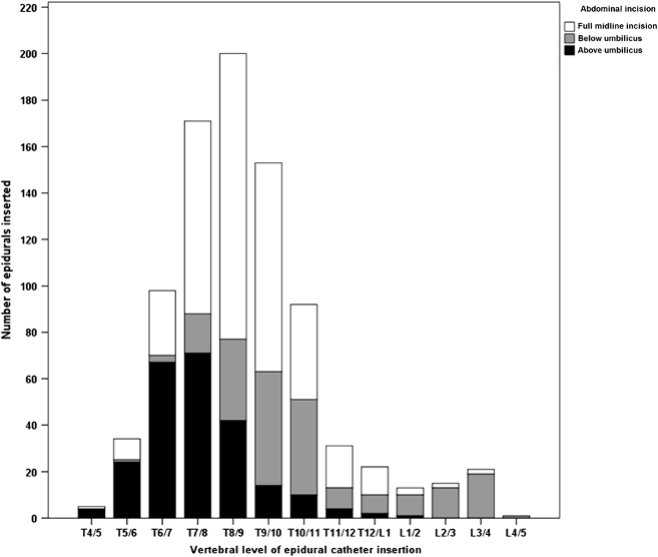
**Number of epidurals inserted at each vertebral level, by extent of abdominal incision, during the study period.**

In total 881 epidurals were inserted between February 2010 - February 2013 for postoperative analgesia following abdominal incisions, with 9871/9987 (98.8%) data fields filled. Median age of the cohort was 66 years (interquartile range, IQR, 57–74) with 60.6% female. Most epidurals were inserted for colorectal (389, 44.3%) or upper gastrointestinal surgery (183, 20.8%, Table 1), with the majority inserted in the thoracic region (819, 94.1%, Figure 1).

**Table 1:** **Factors effecting epidural failure at 48 and 96 hours**

	Number	%	48-hour failure rate (%)	Odds ratio of failure vs remainder of cohort (95% confidence interval)	P value	96-hour failure rate (%)	Odds ratio of failure vs remainder of cohort (95% confidence interval)	P value
**Gender**								
Male	347	39.4	25.6	0.82 (0.60-1.11)	0.21	37.5	1.33 (1.00-1.77)	0.06
Female	534	60.6	29.6			31.1		
**Age**								
≤65	420	48.5	30.7	1.42 (1.05-1.92)	0.02	38.6	1.54 (1.16-2.05)	<0.01
>65	446	51.5	23.8			28.9		
**Surgical specialty**								
Colorectal	389	44.3	28.0	1.11 (0.82-1.50)	0.49	34.4	1.10 (0.83-1.14)	0.52
Upper gastrointestinal	183	20.8	20.2	0.71 (0.48-1.04)	0.07	28.8	0.77 (0.54-1.11)	0.16
Urology	161	18.3	26.1	0.95 (0.64-1.40)	0.78	34.2	1.05 (0.73-1.51)	0.78
Vascular	73	8.3	31.5	1.30 (0.77-2.19)	0.32	36.1	1.17 (0.71-1.94)	0.59
Gynaecology	67	7.6	34.3	1.50 (0.88-2.55)	0.14	34.8	1.08 (0.64-1.83)	0.77
**Level of incision**								
Above umbilicus	250	28.4	21.3	0.65 (0.46-0.92)	0.02	29.9	0.79 (0.57-1.08)	0.15
Below umbilicus	210	23.8	33.0	1.45 (1.03-2.03)	0.03	36.4	1.17 (0.85-1.63)	0.34
Both	421	47.7	27.2	1.06 (0.78-1.42)	0.72	34.4	1.07 (0.81-1.42)	0.63
**Seniority of anaesthetist**								
Consultant	670	77.2	25.6	0.73 (0.51-1.03)	0.07	31.7	0.68 (0.49-0.95)	0.03
Locum	72	8.3	36.6	1.60 (0.96-2.65)	0.07	47.9	1.92 (1.18-3.13)	0.01
Trainee	71	8.2	27.1	1.00 (0.58-1.74)	0.99	37.1	1.18 (0.71-1.96)	0.52
Specialty doctor	55	6.3	32.7	1.33 (0.74-2.39)	0.33	34.5	1.09 (0.61-1.93)	0.77

**Figure 2:**
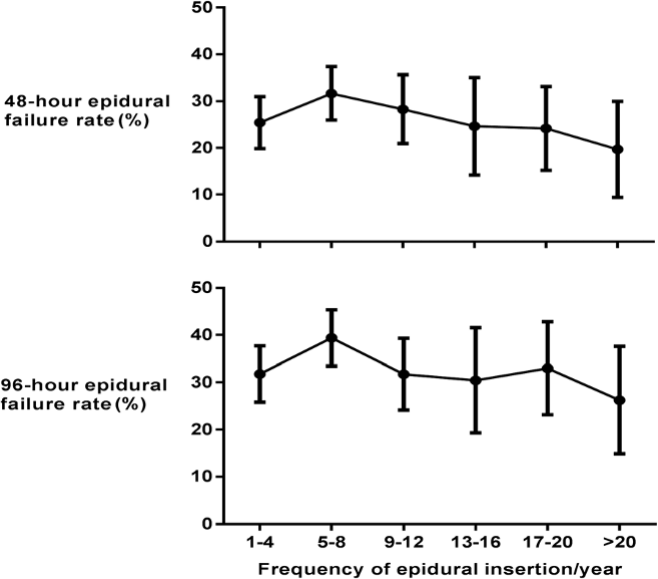
**48 and 96 hour epidural failure rate grouped by number of epidurals inserted per year.**

**Figure 3:**
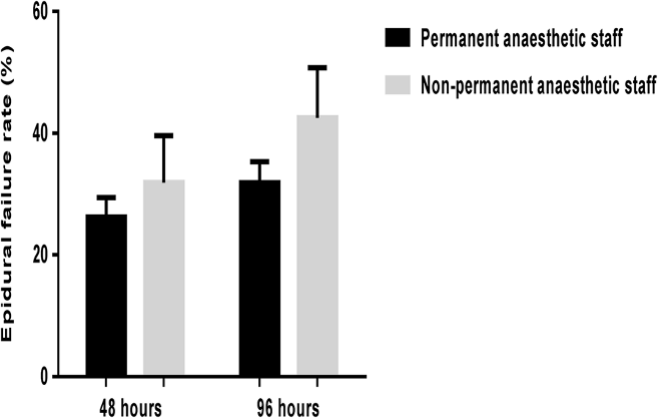
**48 and 96 hour epidural failure rate by permanency of staff (permanent staff defined as consultants and specialty doctors, non-permanent staff defined as trainees and locum staff).**

In accordance with our definitions of failure a total of 236 (27.2%) epidurals failed within 48 hours of surgery and 296 (33.9%) by 96 postoperative hours. At 48 hours, inadequate analgesia was the leading cause of failure (192, 82.8%), followed by technical failure (31, 14.4%), and unacceptable side effects (9, 3.8%). These results were consistent at 96 hours. Of the 881 epidurals inserted, consultant anaesthetists sited 670 (77.2%), with a median of 8 (IQR 4–13) epidurals inserted per anaesthetist per year.Increasing frequency of epidural insertion did not show a significant decrease in failure rate at either 48 (p = 0.36) or 96 hours (p = 0.28), (Figure 2). However, long-term survival of epidurals at 96 hours was greater if inserted by permanent rather than temporary members of staff (non-permanent 60/141, 42.6% vs permanent 228/715, 31.9%, OR 1.58 (CI 1.09-2.29) p = 0.02, Figure 3).

There was a relationship between reported vertebral level of epidural insertion, level of the surgical incision, and likelihood of successful analgesia. At 48 hours epidurals for patients with an incision above the umbilicus failed more commonly if the epidural was inserted below the T9/10 level rather than at or above this level (41/94, 43.6%, vs 123/550, 22.4%, OR 2.69 (CI 1.71-4.23), p < 0.01). Similarly epidurals inserted for incisions below the umbilicus were more likely to fail if placed below L1/2 rather than at or above this level (18/33, 54.5% vs 48/170, 28.2%, OR 3.05 (CI1.42-6.54), p < 0.01). These findings were consistent and again significant at 96 hours for above umbilical (44/94, 46.8%, vs 164/550, 29.8%, OR 2.07 (CI 1.33-3.23), p < 0.01) and below umbilical (20/33, 60.6% vs 53/170, 31.2%, OR 3.40 (CI 1.57-7.34), p < 0.01) incisions.

Patient demographic factors also impacted upon likelihood of epidural success with failure more common in the under 65’s at both 48 hours (under 65; 129/420, 30.7% vs aged >65; 106/446, 23.2%, p = 0.02) and 96 hours (under 65; 162/420, 38.6% vs aged >65; 129/446, 28.9%, p < 0.01, Table 1). Gender, however, conferred no statistically significant increased risk of failure at 48 hours (Women 101/341, 29.6% vs men 135/527, 25.6%, p = 0.21) nor 96 hours (Women 128/341, 37.5% vs men 164/527, 31.1%, p = 0.06, Table 1).

Regardless of whether epidurals were removed as part of planned care or following epidural failure, subsequent 24-hour analgesic requirement was significantly higher if epidurals were removed in the first 24 hours postoperatively (median 67 mg, IQR 32–67) than if removed after this time point (25–48 hours median 30 mg, IQR 10–34, 49–72 hours median 20 mg, IQR 5–40, 72–96 hours median 20 mg, IQR 5–40, >96 hours median 20 mg, IQR 8–43, p < 0.01).

## Discussion

In the absence of published guidelines from the Royal College of Anaesthetists, the Association of Anaesthetists of Great Britain and Ireland, or American Society of Anesthesiologists, it is unclear what degree of ongoing practice is required to maintain competence in providing epidural analgesia. This analysis is, to our knowledge, the first to assess the relationship between epidural failure and frequency of epidural insertion in a general surgical population. Recently published guidance from the European Board of Anesthesiology [18] and the American Society of Regional Anaesthesia [19] outline the requirement for training in epidural catheter insertion, but do not specifically address the frequency with which catheters should be inserted in order to maintain competence.

Our data suggest that increasing frequency of practice does not decrease epidural failure rates. This suggests that epidural catheter insertion may be a skill akin to riding a bike, that once learnt is rarely lost. This finding, coupled with the observation that failure was more common in non-permanent members of staff, implies that knowledge and familiarity with institutional factors may be more relevant in predicting epidural failure than simply inserting a large number of catheters. This view is supported by previous research which reported that as few as 35% of patients with a failed epidural had epidural catheters located outside of the epidural space on computed tomography imaging [20].

This evaluation revealed an epidural failure rate of 27.2% at 48 hours, and 33.9% at 96 hours, which are commensurate with other published case series following intra-abdominal or thoraco-abdominal surgery [3, 4, 8, 20–24]. We report both 48 and 96 hour failure rates, on the basis that Enhanced Recovery After Surgery (ERAS) protocols suggest removal of epidural catheters at around 48 hours [25], while few procedures require epidurals to remain in situ beyond 96 hours. The 96 hour failure rate was higher (42.6% vs 31.9%) when epidural catheters were inserted by non-permanent members of staff compared to permanent members of the department, a finding not replicated at 48 hours.

Providing successful post-operative epidural analgesia is dependent on numerous technical and non-technical factors, beyond simply inserting a catheter. These include: correct epidural catheter placement at an appropriate vertebral level, adequate catheter fixation, and administration of an appropriate local anaesthetic, with or without analgesic adjuvants. Once returned to the ward, patients must be cared for by staff experienced in caring for patients with indwelling epidural catheters, with appropriate patient observation and monitoring. Increased failure rates amongst non-permanent members of staff lends support to the hypothesis that providing successful epidural analgesia is more nuanced than just successfully delivering a technical procedure. Indeed practices which are open to local variation, such as catheter fixation technique and timing of post-operative patient mobilization may more markedly influence epidural outcome and account for the differences seen between permanent and non-permanent staff. The development of standardized local protocols may help to improve success rates for temporary members of staff. In addition, psychological factors may influence the development and severity of post-operative pain, including pain catastrophizing, anxiety, and the degree of pre-operative pain [26]. The data collected for this study did not allow quantification of these factors.

The other major determinant of epidural success was the vertebral level at which the catheter was inserted. For supra-umbilical incisions (that is, above the T10 dermatome) a significantly increased failure rate was seen for catheters sited below this level. Likewise, a significantly higher failure rate was seen if lumbar epidural catheters were used for infra-umbilical incisions, where the incision will cross the low-thoracic dermatomes. This suggests that for optimal epidural efficacy, the catheter should be inserted at a vertebral level corresponding to the cranial extent of the incision, correlating with previous experimental data [27].

The main strengths of our study lie in the size of our population sample and the prospective nature of the data. Whilst several authors have reported series of thousands of patients [21, 24, 28], most other authors reporting on patients undergoing intra-abdominal surgery have analysed smaller series [3, 4, 8, 20, 22, 23]. Additionally the data we present is likely to be reliable, due to the contemporaneous nature of its recording by the staff caring for the patients.

Our study also suffers from limitations. Firstly data were only captured for epidural catheter insertions in the National Health Service for surgical procedures. It is possible that practitioners may perform additional epidural insertions in other settings, for example for obstetric analgesia, in chronic pain management, or in the private sector. Likewise, trainees rotating through our institution may insert many catheters in other hospitals. The degree of experience of practitioners is also not quantified in this study. It is possible that an experienced trainee may have inserted significantly more catheters than some consultants prior to commencing work at the Royal Derby Hospital.

Secondly, this analysis only looked at patients in whom epidural analgesia had been successful established; patients in whom catheter insertion had proven impossible, or in whom analgesia was not successfully established in the recovery room, were not captured. It may be that these factors are related to the number of catheters a practitioner inserts.

Thirdly, as stated previously, the data used for this study were collected for the purposes of clinical care, rather than for subsequent data analysis. Whilst the data are likely to be accurate, we were restricted in the factors we could analyse by the data available to us.

Fourthly, the majority (77%) of insertions were performed by consultant anaesthetists, with much smaller contributions from other seniorities of anaesthetist. It is possible that this disparity has skewed the results and introduced a type I error into the comparison between staff groups. However, we feel that 143 epidural catheter insertions captured in the non-permanent group still represents a reasonable sample size from which to infer a failure rate, and is comparable to several other papers reporting on this topic [20, 22, 27, 29].

Finally, there may be a higher frequency of epidural insertion at which there are demonstrable survival benefits not captured by our study. This study was, however, performed in a major teaching hospital which is in the top 15% of UK Hospital Trusts by number of beds, and it is questionable whether practitioners are reaching higher frequencies of insertion at other centres.

## Conclusion

This study demonstrates that failure rates for postoperative epidural analgesia in major surgery are not dependent upon the frequency with which practitioners insert epidural catheters, but are dependent on permanency of staff. These findings are significant when placed in the context of the GMC’s requirements for clinicians to maintain competence in their clinical practice, suggesting that institutional factors may have greater bearing on epidural success or failure than frequency of task performance.

## Abbreviations

CI: 95% confidence interval
ERAS: Enhanced Recovery after Surgery
GMC: General Medical Council
IQR: Interquartile range
SD: Standard deviation.
